# Book Reviews

**Published:** 1825-04

**Authors:** 


### CRITICAL ANALYSIS
OF
### ENGLISH AND FOREIGN LITERATURE,
RELATIVE TO THE VARIOUS BRANCHES OF
### Medical Science.

Quæ laudanda forent, et quæ culpanda, vicissim

Illa, prius, cretâ; mox hæc, carbone, *notamus*.—PERSIUS.

### DIVISION I.

### ENGLISH.

ART. I.—*An Essay on Curvatures and Diseases of the Spine, including all the Forms of Spinal Distortion; to which the Fothergillian Gold Medal was awarded by the Medical Society of London, and presented, at a special General Meeting, on the 3d of May*, 1824; *with some Additions.* By R. W. BAMPFIELD, Esq. one of the Surgeons to the Royal Metropolitan Infirmary for Children; Fellow of the Medical Society of London; Author of "an Essay on Hemeralopia, or Night-Blindness," of "Practical Treatises on Tropical and Scorbutic Dysentery," &c.—Longman and Co. London, 1824. 8vo. pp. 387.

[Concluded from page 238.]

WE resume our analysis of Mr. BAMPFIELD's work at the SIXTH CHAPTER, containing *the history and symptoms of incurvation of the spine*. This chapter occupies but a few pages, and is in every respect the least satisfactory in the volume: indeed, there are some points which we are not quite sure that we understand, and others that may admit of some difference of opinion; but, as we have professed to give our author's opinions without obtruding our own, we shall, without further preface, state, in as short a compass as possible, his views upon this subject. Incurvation of the spine, according to our author, is rarely attended by those serious consequences ensuing from other curvatures; neither is it accompanied with any remarkable derangement of the nervous influence, but is more generally followed by lameness of the extremity, or psoas abscess. The dissection of these cases shows an increased thickness of the anterior parts of the vertebræ. Our author says, that the lumbar or lower dorsal vertebræ are sometimes incurvated by the gradual contraction of the flexor muscles of the thigh, in consequence of the existence of lumbar or psoas abscess; and immediately afterwards he observes, what must indeed be obvious to every one, that in such cases the incurvation is only an effect of another disease. But the whole of the observations in this section, as we have before said, do not appear to us so clearly stated, or so perspicuously explained, as the former and latter portions of the volume.

We find, under the head of *treatment*, that the dorsal horizontal posture is strongly recommended in cases of this description, where the dorsal or lumbar vertebræ are the seat of the incurvation. If it be situated in the cervical vertebræ, gentle extension may be used; and this may be either temporary or permanent. The collar, or Dr. Darwin's steel bow, may be used for this latter purpose.

"Proper position will also contribute to the recovery, by taking off the superincumbent weight and pressure of the head, and by assisting in keeping the cervical vertebræ extended. For these purposes, the patient should have his head fixed in a socket let into the plane on which he lies, with his chin bent on the chest; and extension of the cervical vertebræ should be resorted to, immediately before the occiput is placed in the socket; the plane should also have a slight angle of inclination at first, that may be afterwards increased. For, the head being fixed, its weight does not press on the vertebræ, even when the patient lies on an inclined plane. Friction and shampooing should be employed. Leeches and blisters may be prescribed, if any pain or inflammation arise. Appropriate remedies should be adapted to the different constitutional disorders that may accompany incurvation, similar to what have been recommended in excurvation. Tonics are in general indicated." (P. 162.)

We think the following remark of considerable importance in the treatment of these chronic affections, and therefore beg to draw the reader's attention to it.

"It may be proper to observe in this place, that the species of temporary incurvation of the lumbar vertebræ occurring during the existence of psoas, or hip-joint abscess, may be sometimes prevented by resisting the tendency to a permanent contraction of the flexor muscles. This is to be attempted by confining the patient to the horizontal posture, and keeping the lower extremity, on the side affected, extended by mechanical means, if necessary, if he lie on his back: but, if he lie on his face, the flexor muscles of the thigh will be extended, in a great degree, by position and the weight of the body and extremity.

"But it is to be regretted that many children fret and cry, and oppose this recumbent plan so effectually, that it cannot be carried into execution, and the object of the surgeon and parents is frustrated. I have seen contractions of various muscles induced in many cases from different causes, all of which eventually yielded to mechanical extension, friction, exercise, and the warm bath or fomentation; but I have not yet seen a case where a contraction of the flexor muscles of the thigh, of *long duration*, has been overcome by those means; a fact which forcibly shows the importance of prevention, as the contraction causes permanent lameness and disfigurement." (P. 162, 163.)

Chap. VII.—The seventh chapter, as it is one of the longest, so also is it one of the most important of the work: it treats of *lateral curvature* as an idiopathic disease, occurring almost universally during *the growth* of the body. Mr. Bampfield thinks the curvature as often takes place on the left as on the right side, in which he differs from Mr. Ward; a difference of no practical moment, and the accuracy of which must be settled by future observers. After enumerating the first symptoms of the approach of this disease, our author goes on to observe, that, when the lateral curvature is once formed in the dorsal portion of the spine, the centre of gravity of the body being lost, the subsequent curves of the spine become the necessary consequence of the first deviation; the number of curves in the last stage being three. This complaint is one of slow growth; but, if the patient should happen to be assailed by any acute disease productive of great debility, the progress of the disease usually becomes very rapid.

We pass over our author's enumeration and description of the attendant symptoms of the lateral curvature, since they have been of late so repeatedly before the medical public, and proceed to the second section, in which the *remote causes* of the disease are enumerated. Mr. Bampfield places rachitis at the head of this list, and we think with great propriety;—following this, we find unequal length of the lower extremities; carrying weights on one arm; bad position of the body; rheumatism of the dorsal and intercostal muscles; abscess on one side of the neck or trunk; scrofula, and tumors. In conclusion, we find that our author first questions, and then denies, that unequal action of the dorsal muscles ever operates as a cause of lateral curvature.

In combating Mr. Ward's opinions on the effect of the more constant use of one arm, in exciting this species of curvature, we conceive that our author has, to a certain extent, been successful; whilst he admits the possibility of the disease being induced by the too early or too frequent use of the muscles of one side, as where young females are employed as nurses, &c. Of the powerful influence of malposition in producing lateral curvature, too many instances may be found in society to render it necessary for us to insist upon it. Nearly the same remarks apply to rheumatism and abscesses, either in the neck, thorax, or back.

After arguing against the doctrine that unequal action of the dorsal muscles operates as a frequent cause of lateral curvature, our author attacks, with great force and truth, the system of miserable restraint introduced into most of the establishments of female education in this kingdom; regulations equally contrary to reason, to health, and (we were going to add) to morals. In this digression we were, however, surprised to find the absolute and unequivocal condemnation of the gymnastic exercises, lately introduced, under the direction of Captain Clias, at the Military Asylum. Surely, Mr. Bampfield cannot mean to put these athletic and manly exercises upon the same level with the miserable trammels which fashion has imposed upon the physical education of the female; nor can he be aware of the strong and forcible instance which the instructor above named presents, in the force and energy of his own muscles, of the successful application of his mode of teaching. We do not mean to assert that his system may not be *too systematic;* but it must be remembered that the bravest and most enlightened nation of antiquity laid more than usual stress upon the exercises of the gymnasium.

Section the third treats of the *immediate causes* of lateral curvature, which will be found in the morbid alteration of structure of the sides of the spinal column. This alteration is rarely the effect of scrofulous caries, or ulcerative absorption, on one side of the bodies of the vertebræ; and, when it is produced, the curvature is generally single, and only affecting one or two vertebræ. (P. 193.) The ordinary lateral curvature, however, is the product of progressive absorption, or of an undue growth of bone, including generally several of the vertebræ. Mr. Bampfield again mentions rachitis as frequently producing this effect upon the spine; and next describes what he has most generally found to be the origin of this species of curvature in the course of his practice,—namely, a disproportionate growth of one of the lower extremities. This disproportion of length he has found to vary from half an inch to two inches and a half, and arises either from an increased growth of one limb or a diminished growth of the other, or from a bending of the knee or ankle joints, causing one to be shorter than the other; but the former is the more frequent cause of curvature. From this, one of two circumstances must occur,—either that the person shall become knock-kneed or bowed in the leg, or the spine become curved. When this disproportionate condition of the spinal column is produced, the lateral curvature may continue to increase, independently of any malformation of the lower extremities; and, during its progress from the slight to the extreme degree of curvature, it frequently happens that the bones forming the knee-joint of the longer extremity become bent outwards or inwards, by which its length is reduced to that of the other, and the spine might be restored, did not the cuneiform shape of the bones now prevent this alteration in the extremities being useful. Our author remarks, that, in his experience, he has found the right extremity generally to be the longest, and, consequently, the great curve of the dorsal vertebræ to be on the left side.

We pass over the fourth section, which contains only descriptions and dimensions of several specimens of the disease, and proceed to the treatment; pausing, however, at page 203, to extract the author's method of ascertaining the degree and course of the curvature, when they are not sufficiently obvious to the eye or finger of the surgeon. In that case—

" 'A plumb-line may be suspended opposite the middle of the occiput, whilst the patient stands in the first position of dancing-masters, which will hang perpendicularly to the centre of the pelvis, and the deviations on each side of this line can be marked with red or black ink.' Having thus ascertained the degree and form of the curvature, we may endeavour to discover what changes can be effected in the distorted position of the vertebræ, and what approximation can be made to the true spinal line. Let the patient first raise himself as erect as possible; he should then perform flexion and extension of the vertebral column; the spine should next be stretched, in the recumbent posture, by assistants; and, lastly, let him be suspended by the head, with weights to his feet, in the manner already described at page 146. If the curves be diminished by these evolutions; if the spine be brought by them to a line nearly straight; if all the vertebræ change their position, and perform some degree of motion,—it may be inferred there is no anchylosis. If no pain be excited by these movements, the absence of inflammation may be deduced, and the prognosis may be favourable. But, if the lateral distortions cannot be lessened by these evolutions and mechanical contrivances, the favourable issue is uncertain, and the patient should not be buoyed up with fallacious hopes of a perfect recovery, that will be most probably disappointed; and, if pain be excited, the prognosis should be guarded, as the termination of spinal inflammation is not always subject to remedial control." (P. 206.)

Chap. VIII. *On the treatment of lateral curvature.*—This may be considered under two heads: first, where the curvature arises from the disproportionate length of the lower extremities; and, secondly, where it arises from any other cause than the above. In the first of these instances, it must be obvious that the surgeon must direct his attention to removing the cause, before he can have any influence over the spinal affection; and the means recommended by our author are the usual mechanical means, aided by frictions, baths, and such internal treatment as the condition of the general health may appear to demand; at the same time adopting the horizontal position; or, if that of sitting be preferred, the spine may be balanced by a weight worn on the head, as recommended by Mr. Grant and Mr. Wilson. To these means other modes of exercise may be joined, and all those which can be performed whilst lying down. It is obvious that in these cases no good can be expected from the use of issues or setons.

When the lateral curvature arises from carrying a weight on one side, the treatment is equally simple. The practice that has produced the deviation must be abandoned; the plan of carrying a weight on the head must be adopted; both arms must be freely exercised, and military drilling may be resorted to. Tonic medicines, nutritive diet, regular rest, and exercise, will of course contribute to the recovery. The application of this principle of cure is easily made in the case of curvatures arising from malposition, or from a habit of always sleeping on the same side. When, however, lateral curvature arises from an irregular growth of bone, exercises in the erect position are not to be attempted. Our author, in such cases, always adopts the recumbent posture, together with extension of the spine used daily; pressure being also used upon the projecting portions of the ribs, and on the spinous processes, in such a direction as to restore them to their natural situation. Relative to the positions to be observed in these cases, Mr. Bampfield explains himself as follows:—

"There are two positions for common use, of almost equal advantage, and they are both obliquely lateral, on a horizontal plane or couch, made for the purpose, and covered with a mattress: hence the patient should be directed, either to lie obliquely on the side on which the ribs protrude backwards, or, in plainer words, to lie on the hump, as the superincumbent weight of the body bearing on this projection will be equivalent to a certain degree of constant pressure, that tends to press those ribs forwards, and to direct the convexity of the spine towards the spinal line; or he should be enjoined to lie on the face with an oblique direction to the opposite side, so that a dosser of lead, modelled in some degree to the shape of the distorted ribs or hump, and varying in weight according to age, or a bag of shot that will assume the turn of the ribs, may be laid on the projection, so as to maintain a constant pressure, with the same indications as in the last position.

"When the curvatures are ambilateral, the positions may be changed from one side to the other." (P. 220.)

The modes of exercise recommended by our author, whilst the patient is in the recumbent position, is in unison with the plans of Messrs. Ward and Shaw.

In the majority of cases of lateral curvature, the medical treatment has been the same as if the patient had been affected with ricketty growth in any part of the body; and, if pain arises in any part of the spine, local bleeding and blisters may be employed. Such are the principal curative means pointed out by our author.

The chapter contains also some remarks in defence of the employment of spinal and other instruments, which are characterised by sound sense, and illustrated in a very perspicuous and satisfactory manner. There are also some controversial remarks, elicited by a passage in Mr. SHAW's work, relative to the exercise of the muscles in this disease; but which we do not feel it necessary to dwell upon, as we believe that, practically, there is little difference in their plans of treatment.

Chapter IX.—is devoted to the *history and symptoms of the angular projection;* a form of disease frequently met with from three to twelve years of age, though occasionally occurring later in life. We shall be very concise in our extracts from this chapter, since the treatment of this complaint is pretty well understood; and our author, with the exception of the facial horizontal position, does not recommend any other means of cure than those usually adopted. It is in this complaint that the utility of setons and issues is universally acknowledged; a practice which also receives the sanction of Mr. Bampfield.

This chapter contains several well-detailed and instructive cases, as well as some accurate accounts of the appearances of the diseased parts on dissection.

Chap. X.—The subject of *dislocations of the vertebræ* is discussed in the tenth chapter; but we cannot deduce any very satisfactory conclusions from the conflicting statements upon this class of accidents. They are, at any rate, of rare occurrence, and very difficult to be remedied, excepting immediate aid be at hand. Our author gives us no rules by which reduction is to be accomplished; and a great portion of the chapter is occupied in combating the opinions of Dr. E. Harrison, and criticising the discordant accounts given of the diseased appearance in the case of a boy named Pratt. A few lines are devoted to the treatment of dislocations of the os coccygis.

The second section of this chapter treats of *fractures of the vertebræ.* Our author recounts the symptoms usually accompanying these terrible accidents, with all the discriminating marks by which the surgeon may be led to suspect the part of the spine where the injury has been received. A knowledge of anatomy will be essentially useful to the practitioner in the consideration of these accidents, as the situation of the fracture not only materially influences the motion of the muscles situated below it, but also will account for the more speedy extinction of life in some instances: as, for example, if the fourth or fifth cervical vertebræ be fractured, respiration will be more affected, and death will more speedily ensue, than when the fracture has taken place lower down. Many modifications of the symptoms will readily present themselves to the practitioner, from a consideration of the part of the spinal column in which the fracture has taken place. Our author observes, that mere concussion of the spinal marrow has sometimes been attended with as much paralysis as fracture with displacement, and a distinction between them becomes difficult; but in such cases there is no displacement, and pressure upon the spinous processes causes no particular pain.

It is scarcely necessary to say, that the prognosis in fractures of the spine is almost always fatal. Where there is no displacement, life is often prolonged for a considerable time; and, in the case of gun-shot wounds, recovery is not impossible. It may generally be said, that, in fractures of the cervical vertebræ below the origin of the phrenic nerve, a week is the extent to which life is prolonged. In the case of either the dorsal or lumbar vertebræ being broken, the period is much longer; and our author gives us a wide range, from four or six weeks to nearly a year.

It does not seem likely that much benefit should, in these cases, be derived from surgical means; excepting by the employment of local bleeding, fomentations, purgatives when necessary, by observing absolute motionless rest on a fracture-bed, and carefully and regularly drawing off the urine by the catheter. This meagre account of the remedies to be resorted to in these accidents leads our author next to consider the proposed operation of trepanning the spine, which has already been performed unsuccessfully in two or three cases, and which has given occasion to a pretty warm discussion between two eminent teachers in this town. In our review of Sir A. Cooper's work on Dislocations, we took occasion to state our opinion as to the inutility of such an operation, from which we argued that no good could possibly be expected. We are happy to find our opinions confirmed by Mr. Bell's authority, as well as by the author before us. We conceive the arguments adduced by Mr. B. to be unanswerable, and we really do not see why a plain rational statement of objections to a particular line of practice, should have caused so warm a discussion between the two eminent teachers above mentioned.

Chapter XI. *Of concussions and stretching of the spinal marrow.*—The symptoms attendant upon these accidents are not dissimilar to those of fracture, from which it is not easy to be distinguished, if there be no displacement of the bone. The prognosis in concussion of the spine should always be a guarded one, since death has ensued even from very slight accidents of this description. Recovery in these cases is either perfect or imperfect. In some cases, paraplegia, with incontinence of urine, has been the consequence. Inflammation, and its consequences, are most to be apprehended from concussions of the spine; though dissection has also shown extravasation of blood, in one or more clots or patches, as well as rupture of all the membranes, allowing the medulla spinalis to protrude through the torn parts, like a hernia.

The indications of cure are, first, to subdue inflammation; and, secondly, to restore the paralytic state of the organs and extremities. To fulfil the first intention, bleeding, the warm baths, fomentations, and opiates, should be employed; the strictest attention to rest and to regimen must be observed, taking care to keep the bowels open: topical bleeding and blistering are also useful; and, after the inflammatory stage is over, frictions, electricity, and tonics, are our chief dependence: but it is necessary to be cautious not to employ these means until we are thoroughly satisfied that inflammation is subdued. In the event of paraplegia continuing for a length of time, the insertion of a caustic issue to the painful part of the spine has been found serviceable. Should the patient continue bedridden for a long period, as in fractures of the vertebræ, sloughing of the soft parts may occur.

Chapter XII.—This chapter is devoted principally to the consideration of *spina bifida*, or *hydro-rachitis*, which is a congenital disease, and, as such, must be distinguished from that more rare disease, dropsy of the spinal canal, which is not attended with a tumor, and may take place at any period of life. We need not stop to describe the appearance of spina bifida: indeed, our extracts from this chapter will be but concise, and chiefly directed to the plan of treatment which has been employed successfully in four instances, by Sir A. Cooper, and subsequently by Messrs. Hickman and Pearson. The mode of treatment in these cases is either palliative or radical: among the former are different kinds of bandages, or plasters, so as to make a gentle pressure on the part, or the application of a properly-adapted truss, to prevent the descent and enlargement of the tumor. The radical methods of cure consist in producing absorption by pressure,—in evacuating the fluid,—or, in those cases where the tumor has a small base, in tying it with a ligature. All these various methods have been found unsuccessful, excepting that of evacuating the fluid by means of a needle; and the result of this operation has been favourable but in a very few instances, and those but of very late occurrence. One of these fortunate cases was published by Sir A. Cooper, in the Medico-Chirurgical Transactions; and our author adds to this the names of three other persons recovered by the same means: one by Sir A. Cooper, the others by Messrs. Hickman and Pearson respectively. The case of Sir A. Cooper's patient, Little, is particularly interesting, since his recovery has been so perfect that he is now enabled, as an apprentice to a pilot, to go through much laborious work, especially in rowing.

A section of this chapter is devoted to *atrophy of the spinal marrow*; but there is nothing certain ascertained as to the history, symptoms, diagnosis, or treatment, of this affection.

We must here draw this long article to a conclusion. There is still another chapter in the work itself upon *inflammation of the spinal marrow;* and, if we do not deem it necessary to make any extracts, it is not because we consider it to be of less importance, or less ably executed, than the former parts of the volume; but it contains little that is not already known, and indeed it could not be expected to be otherwise. The treatment recommended is highly judicious: it may be characterised in a very few words,—it consists in the employment of all those means by which inflammation is to be subdued.

An Appendix, of upwards of thirty pages, contains some very interesting cases of the various forms of spinal disease described by our author in the progress of his work.

---

Art. II.—*Outlines of a System of Medico-Chirurgical Education, containing Illustrations of the Application of Anatomy, Physiology, and other Sciences, to the principal Practical Points in Medicine and Surgery. With coloured Plates.* By Thomas Turner, Member of the Royal College of Surgeons of London, Lecturer on Anatomy, &c. &c. &c.—London: T. and G. Underwood. 8vo. pp. 369.

So many works on Education, of all kinds, have of late proceeded from a prolific press, that it would not be reasonable to expect much matter on this subject substantially novel. The observations may be differently modified,—the opinions may be expressed in different language,—the plans may be arranged with varied order,—and the system of teaching may be diversified; but the science to be taught remains the same. A classical education must consist in learning the Greek and Latin languages, and in reading the Greek and Latin classical authors; the system of teaching may be Etonian, Oxonian, or that of any great school, but the object is unchanged.

The subjects, or objects, of a medico-chirurgical education, must be the sciences of anatomy, physiology, pathology, surgery, materia medica, therapeutics, nosology, medicine, medical physics, and chemistry; and the "Outlines of a System" of such an education, should give a very accurate sketch of all those sciences; should guide the student to the best and easiest mode, in the opinion of the author, of acquiring a knowledge of them; and should point out to the teacher the most useful and engaging method of conveying such knowledge to the student, and of impressing it on his memory.

The title-page of some books very distinctly conveys the precise nature of their contents, whilst that of others bears as little relation to their contents, and gives as little notion of the author's real views, as the title of a play does to that of its plot: so the title of the work under review induced us to suppose that it comprehended in its outline all the sciences appertaining to the medico-chirurgean, (we crave the reader's pardon for so inelegant a term;) and, as it does not embrace so wide a scope, it is but fair to the reader, and justice to Mr. TURNER, to observe, *in limine*, that his system of medico-chirurgical education is not in conformity with the regulations prescribed by the Royal College of Surgeons or the Worshipful Society of Apothecaries, and consequently would not qualify any candidate to be admitted to an examination at either of these courts; that it does not convey any useful instructions to the student in the choice of his books, or in the order of his studies; nor does it often presume to direct the teachers and lecturers in the conduct and plan of their teaching system. The "Outlines of a System of Medico-Chirurgical Education" before us, does not include all the sciences connected with, or indispensable to, such an education; and is, in fact, only a concise and imperfect outline of anatomy, physiology, and parts of animal chemistry, with some illustrations of their application to some pathological and operative points in medicine and surgery.

The work is not formally divided into chapters or sections, but the author discusses his topics in succession, without any necessary connexion. It may, however, be divided into two parts: the first contains three subdivisions, which are entitled "Remarks on Medico-chirurgical Education;" "On the Phenomena of Life;" "On the Human Body." The second is, "On relative Positions, or Medico-chirurgical Views of the Human Body," each article of which is headed by a laconic title,—as "On the Head;" "On the Trunk;" "Abdomen," &c.

From the want of particular arrangement and order, it will not be possible to give an analysis of the contents; to which the variety of the topics discussed would be a further obstacle, the author professing the book to be intended as "an index to the objects which have a claim upon the attention of the student in medicine and surgery."

In his remarks on *medico-chirurgical education*, he observes, "Anatomy is the science of organisation: the means of learning it are dissection and observation. It is the province of dissection to develop the secret springs of action, and to discover the organs which support life; it is, therefore, the foundation of physiology." Entertaining this opinion, he ably vindicates the conduct of anatomists, and adduces strong arguments and proofs of the necessity of dissection; which, as well as all others on the same subject, we would be glad to see submitted to the consideration of those who have, at present, raised and opposed almost insuperable barriers to the obtainment of subjects, and consequently to the due prosecution of the study of anatomy, which, with physiology, "must be considered the basis of pathology, medicine, and surgery."

Mr. T. combats "the too-prevalent opinion, that anatomy is much more useful to the surgeon than to the physician, and thinks Bichat's doctrine of the tissues must convince us of its want of cogency. If anatomy teaches us the intimate structure of organs, the information it gives cannot be over-rated, because it furnishes us with the only means of detecting the existence and nature of diseases; and, but for the valuable facts which have been elucidated *(brought to light)* by post-mortem examinations, the errors of theoretical medicine would have still prevailed, to the great retardation of true science, and the injury of society." * * *

"It may then be deemed a duty to caution the student against so erroneous an idea as that which we have endeavoured to expose. The decree, '*Ecclesia abhorret a sanguine*,' issued by the Council of Tours in 1163, to prevent ecclesiastical characters from performing bloody operations, was the origin of the separation between medicine and surgery; and, under the absurd pretext which this edict conveys, the latter science was cast out from the bosom of the universities, to be practised only by the most presumptuous and the most illiterate of mankind." (P. 8.)

In the article entitled *on the Phenomena of Life*, the author states the nature of the principles of *life* to have been a source of perplexity to the physiologists of all ages, and that, up to the present day, they have only determined its *properties*. He adopts, with some limitation, the theory of Bichat, which regards life as "l'ensemble des fonctions qui résistent à la mort,"—which functions are divided into two classes, those which we have in common with vegetables, being denominated the functions of *automatic or organic life*; and those which are proper to animals, being termed the functions of *animal life*. The author then draws a brief outline of impregnation, of the peculiarities of the fœtal circulation; informs us that, after birth, the economy of the circulation is changed, and all its peculiarities destroyed; and, having noticed the various changes of dentition and growth, concludes by stating that "he has traced life in all its stages, *ab ovo usque ad senectutem*.—We recognise its phenomena," says he, (although, by the bye, none have been described, except those above mentioned;) "but the nature of the principle itself is involved in impenetrable obscurity."

In the division of the work entitled *on the Human Body*, Mr. T.

gives a concise account of the bones, joints, muscles, digestive, absorbent, sanguiferous, respiratory, secreting, nervous, and reproductive *(generative)* systems, and of the organs of sense; in which he blends their anatomy, physiology, and pathology together, with different degrees of minuteness, accuracy, and order. To enable the reader to judge of the ability with which Mr. T. has executed this part of his undertaking, and of the utility of the work, we shall select his account of the digestive system, merely because it is the shortest, and, by extracting the whole, we shall do him more ample justice.

"From the constant actions consequent on organisation and life, and from the continual losses thereby sustained, the body would soon operate its own destruction, if it were not provided with an apparatus to receive the aliment, and to convert the same into a substance capable of repairing the wastes, and of supporting the functions necessary to life. The organs concerned in this important office are called the *digestive organs;* they are, the teeth, salivary glands, œsophagus, stomach, intestines, liver, pancreas, spleen, lacteals, mesenteric glands, and thoracic duct.

"The *teeth* are the organs of mastication, and in the adult thirty-two in number. They are usually divided into four classes,—viz. the incisores, four in each jaw; the canini, two in each jaw; the bicuspidati, four in each jaw; and the molares, six in each jaw.

"Each tooth consists of a base, body, cervix, and one or more fangs; the body is covered over with enamel, a white, vitreous-like, and insensible substance; so hard and indestructible, as to resist the effects of time and circumstances, which destroy every other part of the tooth. It is believed that the enamel is not an organised structure, but a crystallised secretion; for, by feeding an animal with madder, the osseous part of the tooth can be rendered quite red, but the enamel remains unchanged: it is, therefore, supposed not to be subject to the laws of life and circulation. At the extremity of the fang, or fangs, of a tooth, a small hole may be perceived, which leads to a cavity, in which is lodged the pulp, consisting of a gelatinous substance, which supports the vessels and nerves of the tooth. The teeth are confined in their alveoli, or sockets, by means of their periosteum and the gums.

"The *salivary glands* are the parotid, submaxillary, and sublingual; their ducts terminate in the mouth, where their secretion mixes with the food, to facilitate mastication and deglutition.

"The *œsophagus* is the tube which conveys the masticated food into the stomach. It begins at the pharynx, and ends at the cardiac orifice of the stomach; and is a muscular canal, lined by a mucous membrane. Soon after the food has entered the stomach, it loses the character of a masticated substance, and assumes that of an uniform mass. The portion nearest the pylorus is most altered in appearance, for it has taken on the character of chyme; the change having been produced by the gastric juice, which is a powerful solvent.

"The *stomach* is a muscular and membranous bag, not inaptly compared in its figure to a bag-pipe. It has two orifices, two extremities, two curvatures, and two surfaces. Its coats are three,—viz. a peritoneal, muscular, and villous: the gastric juice is secreted by the vessels of the villous coat.

"The muscular tunic has two sets of fibres, a longitudinal and a circular: by the former set, the cardiac extremity can be brought to approach the pyloric; and by the latter, the circumference of the organ can be contracted. It is by these powers that the food is propelled from the stomach, into the first portion of the intestinal canal.

"The *intestinal canal* is divided into the small and large intestines: the small are subdivided into duodenum, jejunum, and ilium; the large into cœcum, colon, and rectum. Like the stomach, the intestinal canal has three coats; but some parts of it have merely a partial investment from the peritoneum.

"The auxiliary organs of digestion are the *liver, pancreas,* and *spleen.* The first of these is the largest gland in the body; the peculiarities of its structure will be mentioned in another place. The pancreas very much resembles the salivary glands, and its secreted fluid is similar in composition and properties to the saliva. The spleen is a spongy organ, composed of cells, in which the arteries terminate: it is supposed to be accessory to digestion, but nothing positive is known respecting it.

"When the food has been partially digested in the stomach, it is carried by the peristaltic action of that organ into the duodenum, where a further change takes place. Into the duodenum open the ducts of the liver and pancreas, by which the bile and pancreatic juice are discharged from their respective organs. There can be no doubt that these fluids are the agents of chylification; although it has been disputed by some physiologists, whether the former has any thing to do with the chylifiant process. The celebrated Blumenbach thinks that the bile separates itself into two portions, a serous and resinous; and that the former probably mixes with the chyle, and is carried back to the blood, whilst the latter combines with the fæces, and acts as a stimulus to the peristaltic motion of the intestines. The ingenious and industrious French physiologist, Richerand, entertains a similar opinion. After speaking of the combined pancreatic and biliary fluids, as serving to penetrate the chyme, to render it fluid, to animalise it, to separate the chylous from the excrementitious part, and to precipitate whatever is not nutritious, he says, 'in bringing about this separation, the bile itself seems to be divided into two parts. Its oily, coloured, and bitter portion passes along with the excrements, sheathes them, and imparts to them the stimulating qualities necessary to excite the action of the digestive tube. Its albuminous and saline particles combine with the chyle, become incorporated to it, are absorbed along with it, and return into the circulation.' How the separation is effected, Richerand confesses his ignorance. He thinks that this act of the animal economy is as mysterious and inexplicable as the action of the brain in producing thought; a phenomenon which many physiologists have considered as exceeding the power of matter. The experiments recently made by Mr. Brodie, and published in Brande's and other journals, are capable of settling the point of dispute respecting the effects of the bile in the process of digestion. Mr. B. was disposed to think that the bile is intended to convert the chyme into chyle, by a chemical change: but, to ascertain whether he was right in this, he completely obstructed the flow of bile into the duodenum, by a ligature on the ductus choledochus. The ligature, and the consequent want of bile, completely and invariably prevented the changing of a single particle of chyme into chyle; a process which takes place at the entrance of the duodenum, and never higher than the pylorus. No chyle could be traced in the intestines, or in the lacteals; but both of these were filled with a fluid like the chyme, which became thicker as it proceeded, and at the termination of the ilium it was quite solid, though not like fæces. The office, then, of the bile is to convert chyme into chyle, and to act as the natural stimulus to the peristaltic action of the intestines.

"When the process of chylification is completed, two functions are set up in the intestinal canal: the one is, absorption of the nutritious part of the food; the other, dejection, or the evacuation of the residuum, or excrementitious portion, per anum. The former is accomplished by the lacteals; the latter, by the vermicular action of the intestines.

"The *lacteals* arise from the inner surface of the small intestines by orifices, not visible to the naked eye in the human subject, and from their origin they pass to the *mesenteric glands.* The lacteal vessels which enter the glands are named, vasa lactea primi generis; those passing out of the glands are called, vasa lactea secundi generis. What change the chyle undergoes in these glands is not known; but they are supposed to add to it a secreted fluid, by which the chyle is more assimilated to the blood. From the mesenteric glands, the lacteals go to the thoracic duct, in which they terminate. This duct begins at or near the first lumbar vertebra; it ascends on the left side of the spine, and ends in the left subclavian vein, at the place where it is joined by the internal jugular.

"Thus, the organs which constitute the digestive system are subservient to various purposes; and on the due performance of each of them depend the nutrition, health, and energy of the body.

"The chyle, or product of the aliment after it has undergone digestion, is found, on analysis, to be composed of water, albumen, fibrine, and saline particles; but there are some differences in the constituent parts and properties of this fluid, as abstracted from animal and vegetable substances. Animal chyle contains more solid materials,—hence is more nutritious; but vegetable chyle is less putrescent,—hence vegetable food is more proper than animal for patients labouring under putrid and some other diseases.

"If healthy and wholesome articles of food be taken, every morbid condition of the chyle must be owing to an undue action of the organs concerned in preparing it. We must, therefore, look to the digestive organs, and rectify their functions, ere the chyle can be improved in its properties." (P. 45—51.)

In a subsequent part of the work, the author adverts to the physiology of *the secreting organs,* in which those of digestion are included, and of which he gives a concise and correct account, as far as the unsatisfactory state of our present knowledge extends. To this he has added the animal chemistry of some of the secreted fluids, and of biliary and urinary calculi; and concludes with some bare and imperfect remarks on their pathology.

In the portion of the work devoted to the *Organs of Sense*, Mr. T. has availed himself of the most modern discoveries, facts, and theories, and has executed this part with considerable industry and ability. In explaining the physiology of those organs, he endeavours to point out to the student the respective shares, or influence, the nerves, muscles, and peculiar structures of each have in the production of each sense; and, to assist him in elucidating the phenomena of vision, hearing, &c. he resorts to the "leading laws" of optics, acoustics, and other branches of natural philosophy. Mr. Turner has also availed himself of some rare cases and facts, of which we select the following, relative to the solvent power of the aqueous humour, and to the power of transmitting sound to the internal ear through the eustachian tube, when the external ear has lost the power of conveying it.

"Analysis teaches us nothing on what the solvent property of the aqueous humour depends, for the most active ingredient it contains is muriate of soda; but that it is endued with this power in an extraordinary degree, is proved by a circumstance which occurred to Mr. Cline, sen. He was operating for cataract; a spasm seized the eye after the needle was introduced, and a portion of it snapped off. Bad consequences were apprehended; but, to the great gratification and astonishment of the surgeon, the fragment of the needle was dissolved, and afterwards absorbed. This interesting fact is mentioned by Sir Astley Cooper in his Anatomical Lectures." (P. 139.)

"I read, not long since, an account of a merchant of Cleves, who became almost totally deaf: one day he happened to be sitting near a harpsichord, while some one was playing, and having a tobacco-pipe in his mouth, the bowl of which rested accidentally against the body of the instrument, he was agreeably and unexpectedly surprised to hear all the notes in the most distinct manner. By reflection and practice, he made a valuable use of this discovery; for he soon learned, by means of hard wood, one end of which he placed against his teeth, to keep up a conversation, and to be able to understand the softest whisper. His son made this the subject of his Inaugural Dissertation, which was published in 1754." (P. 148.)

We now come to the part of the work *on relative Position, or Medico-chirurgical Views of the Human Body*."

The author thus opens the subject by explaining the object of this part of the work:—" After the general description of the human body, which has been given in strict physiological order, it is proper to consider the grouping together of parts; and thus to describe their support and connexions. This constitutes the study of *relative position*,—a subject which has an influence on all the steps which are taken in the planning and performance of the different operations in surgery." Besides this knowledge of relative position, and even besides practice, there is a peculiar firmness of mind and confidence, which are requisite to the performance of an operation with coolness and comfort, (if we may so express ourselves;) for Baron Haller was never able to operate on the living subject, and the great Cheselden, to the last, was generally affected with diarrhœa in contemplating a serious operation.

The medico-chirurgical views are supposed to be taken from parts exposed by dissection, and are described as if the subject for demonstration was lying before us. Nevertheless, Mr. T. by no means adheres to the subject of relative position, or confines himself to those views; but, as inclination prompts or opportunity offers, he describes the anatomical structure of organs or parts, expatiates on their physiology or pathology, and digresses for the purpose of describing accidents and operations, and of giving accounts of diseases, as of phthisis pulmonalis, which are not always perfect; and the treatment of which is not always satisfactorily or decidedly laid down. For instance, under the article *Neck*, which fills twenty-eight pages; nearly the whole of which department is occupied in the history and description of aneurism, of the operations of bleeding from the temporal artery and jugular vein, of laryngotomy, tracheotomy, œsophagotomy, and the extirpation of the thyroid gland, with the history of bronchocele; in the account of the treatment of which, our experience induces us to believe that he has as much undervalued the effects of iodine, and the ointment of the hydriodate of potash, (misnamed by Mr. T. the ointment of iodine,) as he has over-rated the remedial effects of mercury.

"Iodine has recently been extolled, especially by the continental writers, as almost a specific in this disease: it is applied externally in the form of ointment, and ten or twelve drops of the tincture are to be given internally, twice or three times a-day. That friction by any ointment is likely to do good, no one will doubt, as it is a powerful means of exciting the action of the absorbents; but I should place more confidence on mercurial than iodine ointment: and, as absorption may be promoted by combining internal medicines with topical applications, I should have more dependence on small doses of calomel, than on either burnt sponge or iodine." (P. 238.)

As it is one of the principal objects of a reviewer to give the reader an insight into the nature and contents of the work before him, so as to enable him to form his own judgment of the manner in which it is executed, and of the various matters of which it is composed, we will endeavour to analyse the article *Thorax*, which is selected because the author observes, that—

"The thorax, whether regarded anatomically, physiologically, or pathologically, is decidedly the most important part of the human body, as it incloses organs the most essential to our existence. A wound of the head or abdomen may destroy life, but a person seldom survives a penetrating wound of the lungs, in consequence of the hemorrhage which follows; and a wound of the heart is almost instantly fatal. The lungs and heart (strictly speaking) are vital organs; and Nature, all wise in her plans, has taken care to place them in a situation, and to surround them with a wall, well calculated to defend them from external violence." (P. 249.)

Mr. T. then enumerates the constituent parts of the parietes, explains their connexion, and points out the peculiar capabilities of protection they possess for the enclosed contents. The *viscera of the chest* are brought to view by dissection; and, by the aid of a diagram, the course of the pleuritic membrane is sketched out, and its reflections traced with accuracy. The pleura being a membrane of secretion, "an excess of which sometimes causes hydrothorax," Mr. T. is led into the consideration of dropsies, "the proximate cause assigned for which in the different parts of the body, are increased secretion and diminished absorption." He is of opinion, however, that the results of the experiments of Magendie, in the first Number of the Journal de Physiologie Experimentale will have the effect of altering our views relative to effusions.

"We can no longer refer them to either increased secretion or diminished absorption, operating independently. In dropsy from active causes, there is not only an increased secretion, but also diminished absorption; and, to cure in this case, we must bleed. Bleeding has recently been highly extolled, as a remedy in effusions; but it is in effusions from the above causes only that it can do good, when they are of a passive nature; as, in cases where there is a want of energy, it must do harm. In dropsies from debility, an opposite indication is necessary; and, if we succeed in fulfilling it, the accumulated fluid will soon become absorbed, the equilibrium of exhalation and absorption be restored, and the parts again resume their healthy function; but this happy termination can only be expected *when there is no organic disease.*" (P. 255.)

We are then informed that pus and blood may be also effused into the cavities of the chest; and the author decides on the safety of evacuating it in cases of danger, but of its expediency he entertains doubts.

The relative position of the thoracic organs, and the effects of respiration on the parietes and viscera, are next noticed; and, on stating the fact that, "on puncturing the chest, air will rush into the cavity, and then, by the pressure on one side being counterbalanced by pressure on the other, the lungs collapse," he adverts to the experiment and practical deduction made by Dr. Carson, of Liverpool. It has been generally supposed that abscesses of the lungs would be disposed to heal, if they could be kept in a quiescent state: hence, at the recommendation of Dr. Carson, the chest of a gentleman at Liverpool was punctured, and the air being admitted into its cavity, the lungs collapsed; but the practice was unsuccessful.

After alluding to phthisis, punctures of the chest, fractures of the ribs, and emphysema, he leaves the lungs, and continues, "The student will now expose the heart, by making a longitudinal incision in the pericardium." A description of the pericardium, and of the position of the heart, follows; and it is mentioned that a proposition has been made, in cases of hydrops pericardii, by M. Skielderup, of Copenhagen, to remove by a trephine a portion of the sternum between the fifth and sixth ribs, and to let out the fluid by puncture. The course of the aorta, the situation of aneurism and its effects, and the origin of some arterial branches, are now described.

The diseases of the heart are enumerated; of which, it is to be regretted, "with the exception of carditis, we are not in possession of any infallible means of forming a diagnosis:" he, however, attaches some importance to percussion, and to auscultation by M. Laennec's stethoscope; for the mode of using which the reader is referred to Laennec's work, "sur l'Auscultation Mediate," and to Dr. Forbes's translation.

The important nerves of the chest are lastly noticed; and the author sums up by observing, that he "has given the most practical and interesting points in the anatomy, physiology, and pathology, of the structures of the thorax." The reader will probably suppose that the article should have terminated by the observation, that he had now drawn all "the medico-chirurgical views" of the thorax, and laid down their "relative position."

Three articles, entitled *Abdomen*, *Upper Extremity*, and *Lower Extremity*, follow; and the author concludes with the following paragraph:—

"After all his labour, however, he cannot flatter himself that the work is complete in its arrangement, or in its execution; but he submits it to professional candour, and avows his willingness to avail himself of any suggestion that may improve it, or further tend to facilitate the acquirement of that knowledge which has a direct influence on the happiness of mankind." (P. 369.)

Being thus invited to express our opinion, we would observe, that the author has evinced considerable industry, some research, and an ability that, when matured by experience, promises well. The work is, however, deficient in arrangement, and its professed objects are constantly lost sight of; the information it conveys is not so full and particular as is necessary to the student, who has all to learn; and, upon the whole, we look upon it rather as a sketch to be filled up, than as a complete and finished view of the subjects it is intended to illustrate.

---

### DIVISION II.

### FOREIGN.

ART. III.—CARL WENZEL, der Heilkunde Doctor, Geheimerrath, Ritter des Kaiserlich Russichen St. Annen, des Königlich Preussischen rothen Adler und des Concordien; mehrerer Academien und gelehrten Gesellschaften Deutschlands, der Schweiz, Russlands, Frankreichs, Spaniers, und so weiter Mitglied, *über die Krankheiten am Rückgrathe.* Mit acht Kupfertafeln.—Bamberg, 1824.

C. WENZEL, Doctor of Medicine, &c. &c. &c. *upon Diseases of the Spine.* With eight Copper-plates.—Bamberg, 1824.

WE have laid before our readers detailed accounts of the works of Mr. WARD, Mr. SHAW, and Mr. BAMPFIELD, in the order in which they successively appeared; having only concluded our analysis of the last gentleman's Treatise in our present Number. As we may not speedily have an opportunity of again recurring to this subject, which has occupied so much attention during the last few years, we now purpose to take our leave of it for the present, by an account of some of the most important facts contained in the splendid and able work, the title of which stands at the head of this article.

We candidly confess we do not like the size of the volume: a medical book in folio, consisting of 450 pages, is an alarming object to the eye of the most determined student. Every page, however, teems with proofs of the author's extensive reading; and we confess the references to continental works, with which we are totally unacquainted, are very frequent. In the hospitals of Wurzburg, Vienna, Pavia, &c. &c. our author has enjoyed numerous opportunities, during the last thirty years, of collecting experience upon the subject of which he treats. He was induced to pay particular attention to it from the recommendation of his highly honoured preceptor, WEIDMANN whose name is honourably known to the Profession.

The work commences with a very elaborate, and perhaps even unnecessarily minute, anatomico-physiological description of the spine, and the parts connected with it. A series of interesting cases, illustrative of the various diseases of the spine, are also detailed at great length. The opinions of an immense body of authors are contrasted with the results of the author's own observations. The valuable contributions which have been added by English surgeons to this interesting class of diseases, are duly appreciated, and acknowledged with candour.

The sixty-sixth section of the work is entitled "General conclusions from experience, respecting the origin, the seat, and the causes, of curvatures of the spine, which result from inflammation, and ulceration of the vertebræ." We translate the whole of it, for the benefit of our readers. It contains much important matter, which cannot fail to be interesting to every part of the profession. It is written in an aphoristic form.

"1. Wherever we find true pus, inflammation is the cause of that appearance.

"This assertion is in perfect accordance with the testimony of those who have formed a correct opinion of true inflammation. It would be confirmed by all, if many of us had not been accustomed to consider as inflammations a great number of morbid appearances, which are not so in fact.

"2. When we find abscesses connected with this disease of the spine, we always detect true pus in them, in whatever part of the body it may make its appearance.

"3. The changes of the pus which we sometimes find in these abscesses, depend not upon its original formation, but upon many circumstances. We must consider the secretion that will be poured out from the lymphatics which lie in the neighbourhood of the diseased part, and which must take place from the numerous absorbent vessels, that are partially destroyed, when there is a greater or less degree of destruction around the abscesses.

"4. Diseases of the bones arise from causes which either immediately affect them or their periosteum, or both these parts. The vertebræ are acted upon in the same manner, by any causes, as the other bones of the skeleton.

"5. Disease in the vertebræ is productive of the same effect as in any other bony structure of entirely spongy formation.

"6. The distinctions that may occur are similar to those which we find in the spongy parts of the bones in the neighbourhood of the joints.

"7. In these particular diseases of the spine, we must therefore consider the proximate cause to be either inflammation orginating in the external or internal periosteum, the internal ligamentous band, which performs the office of periosteum to the parts it covers, or in the bones themselves.

"8. We possess cases which induce us to believe that the disease may originate from various changes which exist in the longitudinal ligamentous bands, particularly the anterior.

"9. From the examination of bodies, however, we do not possess any information respecting this double origin of the disease, which can be considered incontrovertible. On the contrary, most of these cases have been doubtful.

"10. From some cases we should be induced to infer, that when we do not detect the original seat of the disease in the anterior longitudinal ligamentous band, that changes exist in the posterior ligamentous band, which is extended over the vertebral canal, of a perfectly similar nature to those which occur in the anterior.

"11. The peculiar symptoms of this modification of the disease have been explained by some authors, and deduced from pressure upon the spinal marrow, arising from the swelling of the parts surrounding it.

"12. This opinion is not confirmed by examinations, which have lately been very carefully made; and, indeed, it would be difficult to demonstrate how any cause, capable of producing disease, should act exclusively upon the posterior ligamentous bands and the internal periosteum of the vertebræ, without exerting its injurious effects upon the anterior.

"13. The vertebral canal has, in many respects, so much resemblance to the cranial cavity, that we are justified in drawing analogous conclusions respecting them; that similar diseases of the cavity of the cranium and spinal marrow may be connected with each other, with similar results to the analogous parts,—the brain, the spinal marrow, and the enveloping textures.

"14. In the cranial cavity, we find that great injuries to the pericranium are invariably connected with morbid changes of the dura mater; and, on the contrary, diseases of the dura mater, with affections of the external periosteum of the cranial bones; especially if both diseases derive their origin from violent external causes. This fact is explained by the vascular connexion which exists between these parts.

"15. From the same cause we know that, when a sufficiently violent cause operates upon the vertebral column, we shall find morbid changes of the external and internal periosteum of the vertebræ.

"16. The morbid affections of the spine are generally to be considered as the result of internal causes.

"17. The different thickness of the bony structure of the cranium and the vertebral canal, creates considerable difference in the effects that are produced by causes apparently similar, be they internal or external. We do not, therefore, so frequently witness morbid affections of the external and internal periosteum of the vertebral canal, arising from those causes which most probably would produce them in the membranes of the cranium.

"18. The observations of those who have placed the original seat of the disease in the intervertebral cartilages, are very imperfect and doubtful; although it is true that the inflammation, which is the proximate cause of the disease, may affect that part. From examinations and experience, we have ascertained—

"19. That the inflammation—in the strictest sense, a true inflammation,—takes place in the vertebral bones, and in all those parts which have a natural vascular connexion with the vertebral column; consequently, either in the periosteum or that structure which occupies its place.

"20. That, because in many cases we do not perceive the inflammatory period, we are neither justified in denying its existence, nor in applying the term chronic inflammation.

"21. The cases from which the existence of chronic inflammation has been inferred, are doubtful. Equally doubtful is the assertion, that the inflammation gradually extends from one vertebra to another, until many are affected, as we find in the latter periods of the disease.

"22. It is according to nature and experience to believe that we should find those vertebræ in an ulcerated state, upon which the causes of inflammation originally operated.

"23. That, although we observe in some cases, in consequence of the continued action of the original cause, or of some other adventitious cause, an extension of the disease in the vertebræ, the idea is not refuted that, in the spot originally affected, inflammation had ran its regular course, and passed into suppuration.

"24. The anterior longitudinal ligamentous band we find more or less thickened, and separated from the vertebræ. We find all tendinous parts, which have a more or less firm connexion with bones, separated from them, when they are in a state of ulceration. We find them (the tendinous parts) in a state which does not justify us in positively determining whether the inflammation originally extended from them, or whether the changes we perceive had resulted from inflammation of the bones, which had existed at a very early period of the disease.

"25. According to the nature of the spinal affection, its symptoms, and the effects which are produced upon the patient, we may presume that the disease exists whenever there is inflammation of the vertebræ.

"26. The nature of the disease is not altered by the different extent of inflammation in different cases.

"27. The disease, therefore, may affect a greater or smaller number of the vertebræ. It may attack only their external superficies, or destroy their substance more or less.

"28. Under these circumstances, we shall constantly detect a collection of matter between the anterior longitudinal ligamentous band, and the ulcerated surface of the body of the vertebræ.

"29. From the above observations, it appears that inflammation and ulceration of the vertebræ present the only true rationale of the disease.

"30. Most of the causes of the disease which have been enumerated by the ancient physicians, at least, show that inflammation would be the probable result; and, as far as we can judge, the disease arose from inflammation, although they have not always exactly described any inflammatory affection or ulceration.

"31. Rachitis has been enumerated amongst the causes of this disease; but it could hardly be considered as such by the surgeon who had never witnessed the inflammation and ulceration of the bones arise from rachitis.*

---

* This question has been discussed particularly by Mr. Shaw, who is of opinion that the common cases of spinal deformity are independent of rickets, and that, excepting where the limbs are ricketty, the pelvis is not affected, however great may be the curvature of the spine. (*Vide* Shaw *on Distortion of the Spine.*)

"32. Neither in rachitis, nor during the morbid states which it produces, shall we find an ulcerated state of the bones, which have suffered even from its most violent attacks. Amongst many other proofs of this fact, may be mentioned the dreadful degree of distortion of the spine which may be the consequence of rachitis, without any ulceration of the vertebræ, whatever may be the extent of the deformity.

"33. Rachitis, and anchylosis, which is the consequence of it, appear to be widely separated from inflammation: we should, therefore, never enumerate it as a cause of the disease we are now considering.

"34. Scrofula has been enumerated as one of the exciting causes of this disease; and, as the contrary cannot be proved, it may be admitted. When scrofula acts to a great degree upon the bones, it produces inflammation in them, and all those symptoms which are common to inflammation of the bones in general, and especially of the joints, or of those parts which have an analogous structure to the joints.

"35. The facts which have occurred to me respecting the origin of this kind of curvature of the spine from scrofula, would by no means justify me in denying that morbid poison is sometimes the cause of it. I oppose only, and I trust upon sufficient grounds, the unlimited opinion that scrofula is the only cause of many diseases which we detect in children, and more particularly in the spine.

"36. In opposition to the opinion of those who assume that there is a specific cause for those changes which take place in this disease of the spine, no argument need be adduced. The conjecture carries with it its own refutation. Those who have supported this opinion have admitted, in the strictest sense, an inflammation of the vertebræ as the cause of this curvature of the spine.

"37. We are to consider as a cause of this morbid affection, every irritation, be its kind or nature what it may, which is capable, when it acts with sufficient energy upon the parts which constitute the spine, of producing inflammation there, which will ultimately lead to suppuration.

"38. It is very certain that, even in infancy, this disease arises, much oftener than is imagined, from external violence of various kinds affecting the spine. The apparent insignificance of the cause, or the period which elapses subsequent to its application, and before the appearance of any manifest symptoms, make us attach less importance to it than it really deserves.

"39. Whilst this disease of the spine arises from numerous external causes, and first as the consequence of the destruction of the natural proportion of the parts to each other, the affection of the absorbent system is strongly marked by the symptoms. We are then accustomed to consider scrofula as a cause of the disease. With respect to the causes, however, we are, in fact, much in error.

"40. I believe that these opinions are so true,—so conformable to the nature of the disease, and to all the experience we possess upon the subject, from a careful examination of bodies, that no reasonable argument can be maintained against them.

"41. They who maintain that this is a disease affecting children alone, are mistaken: every age and each sex are exposed to it.

"42. The spongy texture of the bones which are attacked by this disease, renders it difficult, in every period of life, to form a clear opinion of the precise nature of the disease under which the patient labours.

"43. When the disease commences in children, particularly those of a very tender age, it is still more difficult to detect the true inflammation as the cause of the disease, since very suspicious symptoms of a really existing curvature of the spine may occur at that age, without the coincidence of any morbid affection of the vertebræ.

"44. If a curvature has really taken place from inflammation and ulceration of the vertebræ, in a very early period of life, we shall certainly be inclined to look for the proximate cause in inflammation of the parts.

"45. This disease may either affect the cervical, the dorsal, or the lumbar vertebræ, or the os sacrum. It will always be essentially the same, although frequently the external appearances are different,—at least, the general symptoms which accompany the disease."

The next chapter is entitled "General results from experience of the external appearances, and the symptoms, of this disease."

"46. The striking external changes which we observe in the vertebræ, are appearances which we detect in the majority of cases of this kind. These appearances are, in general, more apparent in proportion to the ulcerative destruction of the bones of the vertebræ.

"47. We are by no means, however, to wait for the external malformation of the spine which we observe to result from ulceration of the vertebral bones, in order to be fully convinced of the presence of that disease.

"48. We frequently detect an ulcerative destruction of the bones affecting many of the vertebræ, without any external appearances of malformation, although—

"49. The malformation which we observe in the spine is determined from the extent of the suppuration which we detect in some of them.

"50. The external and evident appearances of deformity of the spine are found to be considerable when but few vertebræ are affected; and on the contrary.

"51. The more evidently the presence of the disease is manifested by the degree of deformity, the more difficult will be the management of the cure: while the less the external form of the spine suffers from the disease, the greater will be our expectation of contributing to the recovery of the patient. But little assistance can as yet be derived from the investigations of those who have endeavoured to decide the symptoms of the disease in its incipient state.

"52. The principal cause which has impeded our progress in this respect, is that we do not feel disposed to admit the existence of inflammation, when it is not manifested by the appearance of all, or the greater number, of its peculiar symptoms.

"53. The external appearances of this disease differ during life from those which we detected in preparations we have had an opportunity of seeing, exhibiting examples of cure in very formidable cases.

"54. Formerly, in order to account for the great and striking changes which we detect in the spine, or in some particular parts of it, when a cure has been effected, a fracture of the vertebræ, arising from external violence inflicted upon the back, was assumed.

"55. This opinion affords a sufficient proof that conclusions were inferred from dried preparations, in which the degree of deformity, and the particular state of the vertebræ which have suffered, are frequently of such a nature as to countenance the supposition above stated;

"56. Which supposition certainly will not arise during life, or from the history of the progress of the disease; for, however extraordinary may be the deformity which we see in preparations of cases in which a cure has been effected, it is certain that during life it arose gradually, and in such a manner as to create surprise that any appearances of recovery should take place; particularly if we had an opportunity of watching the whole progress of the disease.

"57. The incipient symptoms of this disease are of such a kind, that they can only be detected by those who are thoroughly acquainted with the natural construction and state of the spine. To such only will they impart a knowledge of the disease, as they are not very striking, although they must suffice to enable us to determine the existence of the disease.

"58. The symptoms differ, as different parts of the spine are affected.

"59. In the inferior dorsal vertebræ, and in the first or second lumbar, the symptoms are soonest perceptible. In the cervical vertebræ, and the lowest lumbar vertebræ, we shall be scarcely able to detect the existence of the disease by any external signs. When ulceration of the sacrum takes place, it is evident that we cannot expect to ascertain it from an external examination.

"60. We presume, therefore, that every effort must be directed to detect the disease at an early period, rather from its causes and slight symptoms, than from any external and manifest deformity of the spine.

"61. It must be confessed that our knowledge upon this part of the subject is as yet very imperfect; for—

"62. To the external signs of the undeniable presence of this disease belong abscesses, which arise from ulceration of the vertebræ, and which may appear in various parts of the body.

"63. That for a long time we were ignorant of the nature of these abscesses, requires no proof; and that we are still so with respect to to their causes, is shown by daily experience.

"64. Even surgeons, as well acquainted with this disease as Paletta, have imagined that there was some peculiarity in these abscesses, and in the ulcerative destruction which takes place in the vertebræ.

"When these abscesses appear, if there does not exist at the same time any spinal deformity, or a body of other symptoms proving the existence of the disease in question, most surgeons either regard it as a disease of but little consequence, or as a state in which they conceive themselves bound to wait for a still further development of symptoms before they determine its nature. This uncertainty not unfrequently exists until the disease has reached an incurable height.

"66. The situation in which these abscesses appear frequently increases the perplexity, rather than it affords any assistance to the practitioner to determine their true nature. For example, when they appear under the glutei muscles, the case is considered to be an aggravated instance of coxalgia; which is not the fact. Hence we frequently find that the mode in which these abscesses appear, the place they occupy, the time taken in their formation, and many other circumstances, multiply the erroneous conclusions which are deduced as to their origin.

"67. If, therefore, this kind of abscess, as soon as it is once formed, manifests clearly a high degree of the disease in question, we are imperiously called upon to endeavour to ascertain the existence of the disease, of which it is the constant result, at the earliest possible period after the formation of the abscess.

"68. A more perfect description of these abscesses, and of their probable causes, the parts in which we observe them, will be described when I enter upon the symptoms of the disease.

"69. Amongst the symptoms from which it is said we may form some judgment of the existence of this disease, palsy of the lower extremities has been enumerated.

"70. I have made some observations above, which refer particularly to this symptom of the disease, as far as it is detected in the inferior extremities.

"71. I have there stated that it may occur in the upper as well as in the lower extremities, and have recorded the circumstances which give rise to it.

"72. If, for the purpose of positively determining the existence of the disease, we should wait for the occurrence of palsy of the limbs, our opinion will be formed at a very late period of the disease.

"73. It is, in fact, not palsy, in the strict sense of the term, either of the upper or lower extremities, which we require, in order to be perfectly convinced of the existence of this disease. In the progress of the disease, our attention will always be attracted to the deranged functions of other organs of the body, which will perfectly convince us that the nervous influence is destroyed, upon which the performance of those functions depends.

"74. That a paralytic state, and particularly of the inferior extremities, should have been so universally considered as the peculiar symptom of the disease, arises from this circumstance,—that its causes have been deduced from pressure upon the spinal marrow, which was either produced from a morbid enlargement of the vertebræ, an evident morbid affection of the posterior longitudinal cartilaginous band and periosteum which line the canal, or in abscesses pressing immediately upon the spinal marrow.

"75. I think I have sufficiently proved that, in most of the cases which we have an opportunity of observing, none of these causes have existed; and that it is a fair and irrefutable inference, that the paralytic state, which we see as the consequence of pressure upon the spinal marrow, is essentially different from that which we observe as a well-marked symptom of this disease.

"76. The examination of the bodies of those patients who die during the inflammatory period of the disease, or suppuration of the vertebræ, either as a consequence of this affection, or some other accidentally coincident disease, generally affords us but very imperfect information with respect to the condition of the spinal marrow, of the nerves passing from it, and of the parts contiguous to the seat of the disease, as under these circumstances there is generally a sphacelous destruction of the part previous to death, which renders a correct anatomical examination impossible. Hence the appearances upon dissection should not be adduced to support the opinions of those who presume that the greatest part of the symptoms of the disease arise from a morbid affection of the spinal marrow.

"77. In such a state of the body, it is hardly possible clearly to distinguish the condition of the spinal marrow, and more particularly that of the nerves which arise from it, or of the anterior smaller branches which contribute to the formation of the sympathetic nerves, and of their posterior large branches. We generally find the spinal marrow, and the nerves which arise from it, in a state as if they had been examined by the knife, or had been acted upon by a slight degree of maceration,

"78. We should not, in fact, without hesitation, attribute the symptoms we detect in this disease to palsy. The appearances in all the parts which derive their nerves from the affected portion of the spinal marrow, the imperfect use of the limbs, or the diminished voluntary power in different parts of them, must sufficiently enable us to determine the real presence of this disease of the spine.

"79. Palsy, and what (in the strictest sense of the term) we connect with that expression, is an appearance which is commonly observed only in the last stages of the disease, and in such cases in which we are scarcely able to contribute any thing to the cure.

"80. The slighter degree of impaired function of the parts, and the useless state of the limbs, do not entirely depend upon the changes which we observe in the spine, as a result of this disease; for we see them—

"81. In this disease, previous to any alteration having taken place in the spine.

"82. We frequently find the above symptoms, in a very aggravated degree, when the curvature of the spine is improving; and often much less severe when a very considerable deformity of the spinal column is observable.

"83. We do not detect the above symptoms in those changes of the spine which arise from other causes than inflammation and ulceration of the vertebræ: for example, in the highest degree of spinal curvature arising from rachitis; and,

"84. In proportion as the cure advances, all the symptoms of the destroyed functions of the parts will be diminished, whatever may be the degree of deformity which may have been inflicted upon the spinal column.

"85. Experience has taught me, from an examination of many preparations relative to the cause of this disease, that, however great may be the destruction of the bodies of the vertebral bones, the canal for the spinal marrow will be found healthy, and in its natural integrity.

"86. A careful examination of a great number of preparations shows that what the spinal canal loses in length, from the morbid curvature, is added in some degree to its circumference.

"87. This appearance is very clearly shown from plates of many preparations. In the opening between every two vertebræ for the passage of the nerves, there is a manifest increase of circumference, in proportion to the loss sustained by the body.

"82. The origin of the above symptoms must, therefore, be derived from some other cause, and we find it in part in the nerves which arise from the spinal marrow.

"89. That they arise partly from the affection of other nerves, which are contiguous to the spine and the suffering part, must be evident from the nature of the symptoms.

"90. Most of the morbid appearances which we detect, and the latter particularly, depend upon morbid affections of the sympathetic nerves.

"91. That the symptoms may arise from an affection of the nerves which are contiguous to the diseased part, and particularly of the sympathetic, explains to us a train of symptoms which we detect at the commencement of the disease. These are of such a nature that they prove the influence of the nerves upon the different parts, and especially beneath the affected part, is destroyed.

"92. These symptoms are observable before the occurrence of any affection of either the upper or lower extremities. They merit our most careful attention, since the incipient stage of the disease is made known by them.

"93. The anatomical examination of those bodies which had suffered from this disease, seldom gives us any satisfactory information upon the true condition of the nerves during the existence of the disease; because, as I have before observed, such changes precede death as to render a satisfactory inspection impossible."

However anxious we may be to impart to our readers much valuable information contained in this laborious and able work, our limits oblige us to be brief in our extracts of the observations made by the author upon the mode of treatment demanded for the various affections of the spine, which he has treated of in so masterly and faithful a manner. We have purposely avoided breaking the thread of Wenzel's views by any interruption of our own, although in the foregoing statements there are many facts, relative to general pathology, and to the disease in question, which he appears to consider as incontrovertible, that do not entirely square with the opinions entertained in this country.

### *Of the Treatment of Curvature of the Spine, which arises from Inflammation and Ulceration of the Vertebræ.*

*Treatment in the period of inflammation.*—Our practice must be very different in those cases of curvature which arise from inflammation, and those which arise from ulceration of the vertebræ. If ulceration of the parts exists, it can only be considered as the result of inflammation. In order that the treatment we adopt may be successful, it is absolutely necessary to detect the disease in its incipient state. If this stage of the disease should have passed by, and given place to suppuration, our success is doubtful, and in a majority of cases our attempts will be ineffectual. If we are aware that the spine has been exposed to any cause likely to give rise to inflammation, topical bleeding, by means of cupping and leeches, will be required. In most cases, particularly in adults, the former mode of drawing blood deserves our preference. Even in doubtful csses, the application of these means is demanded, although they may appear unnecessary or too heroic. In such cases, the loss of time consumed in the application of a trifling treatment, is irreparable. In adults particularly, and where any external violence has taken place, or congestion of blood from any other cause exists in the vertebral canal, the means above mentioned are particularly indicated. In infancy, at which period curvature of the spine generally takes place, many circumstances may restrain us from commencing the treatment by local blood-letting; although it will, of course, be demanded, if there are manifest signs of inflammation. The treatment recommended by Pott is necessary in such cases.

The nature of the causes from which the inflammation of the vertebræ arises, the number and degree of the symptoms which lead us to apprehend that inflammation may have taken place in any part of the vertebral column, will conduct us, after local bleeding, to the application of external means; by the use of which we can have no other object in view than to produce an irritation upon the external surface of the affected part, greater than that of the inflammation. As in the first period of the disease, we shall seldom have any external marks upon which we can place reliance, it will be judicious to apply counter-irritants upon a greater surface than would be required if we possessed more positive knowledge of the causes of the disease from existing symptoms. A correct knowledge of the nature of the causes which produce inflammation of the vertebræ, leads us much further than to the unlimited use of external irritants. In the commencement of the disease we should not inconsiderately produce external ulceration by moxa, or other means, as it is difficult to determine the precise spot upon which it is required. In such cases, the application of milder irritants to the whole length of the spinal column is more advisable. Of these we possess so many, that it will not be difficult to make a choice. Blisters, tartar-emetic ointment, or (what has appeared preferable in my own practice, in most cases,) the application of tartar-emetic in the form of plaster, upon various parts of the spine, or the acrid plaster recommended by AUTENRIETH to produce small ulcerations in different spots, sufficiently fulfil the indications in most cases. During the inflammatory period, mere powerful external irritants will not be advisable, particularly if there is much pain in the part affected. Even in those cases in which we can determine the seat of the disease, it is injudicious to apply external irritants of any kind too near the affected part. By such treatment we not only increase the local pain, but positively counteract the very purpose for which the application was used. It is proper to apply the artificial irritation at some distance from the part affected.

### *Treatment during the Period of Suppuration.*

When the inflammatory stage has subsided, and deformity of the spine has commenced from suppuration, which is indicated by local appearances, a higher degree of irritation will be required. It cannot be denied that it is to POTT we are indebted for the accurate distinction of those cases in which artificial ulceration should be applied. LARREY* has made some useful observations upon the occasional application of moxa. Many surgeons deny the curative power of artificial ulceration in this disease. CAMPER† gives it as his opinion that the treatment recommended by Pott will not produce the expected good effect. VAN GESCHER coincides with Camper. BOYER maintains that, when an abscess is formed as a consequence of this disease, that artificial ulceration is a useless torment to the patient. The collected experience of a great body of surgeons is at variance with these assertions; if, indeed, we have recourse to external irritants before the disease has advanced so far as to be beyond the reach of any treatment.

There exists much difference of opinion as to the *modus agendi* of these means. By many, the principal benefit from their employment is supposed to consist in the free exit which they afford to collected pus. By others it is thought that the absorbents are excited in the part affected, and that the matter is thus removed. The first conjecture arises from the examination of bodies, in which an immediate connexion has been discovered between the external artificial ulceration and the diseased vertebræ. It has before been stated that this conclusion is erroneous, inasmuch as the sphacelated destruction in the circumference of the part affected, which takes place previous to death, so completely alters the natural condition of the parts, that we may find a connexion between the external ulceration

* *Recueil de Memoires de Chirurgie;* par le Baron LARREY.—Paris, 1821.

† *Prix de l'Academie,* tom. v. p. 2, 288.

and the internal disease, which did not exist during the progress of the disease. In the opinion of our author, the benefit of external irritants does not depend upon the quantity of matter which they daily produce, but upon the degree of irritation. He considers the use of moxa, as recommended by Baron Larrey, preferable to caustic issues or the actual cautery. By whatever means we produce external irritation, we are to guard against giving rise to fever and constitutional disturbance, by the too quick repetition of the remedy. A certain period should elapse between each application. The same remark will apply to the use of these more powerful irritants, which is above stated with respect to the milder class: viz. they are not to be applied too near the part affected.

The last chapter of the work contains some interesting observations upon the subject of the application of instruments.

One of the most interesting questions that at present divides the opinions of surgeons, is the cause of the *high shoulder*,—a species of deformity so common in young females, and particularly those of a high rank in life. We find that our author and Mr. SHAW are at issue upon this point. In Germany it is a prevailing opinion, and it is supported by the authority of Wenzel, that this kind of deformity arises from undue muscular action. Mr. Shaw* opposes this doctrine; which has, however, many able advocates in this country.

Although our notice of this work has run on beyond the limits we usually dedicate to any particular author, it is incumbent upon us, in justice to him and to ourselves, expressly to state that we do not present the foregoing abstract as a perfect review of the numerous and valuable facts embodied in this elaborate production. Whoever is particularly interested in the subject of diseases of the spine, must necessarily consult Wenzel. Every opinion he maintains is supported by highly interesting cases. The *ensemble* of the work presents indisputable proofs of the most determined industry, and of an anxious wish to convey in one volume a perfect knowledge of every part of the subject. It is certainly to be lamented that so much space should be occupied in a detailed anatomical description of the spine and its appendages. We do not look for such information in practical works. In this country, a medical treatise in atlas folio would be rather a novel sight. As reviewers, "the pioneers of literature," we are not particularly anxious to become more familiar with it. It has been truly said, that a pleasing exterior is a letter of introduction to a man: such, to a certain extent, is also the case with books. To many, we apprehend that the first view of the ponderous tome before us would be rather repulsive.

---

* SHAW *on Distortion of the Spine*, p. 66.

He who enters upon its perusal, *ab initio ad finem*, must assuredly "screw his courage to the sticking place," or he will fail; although we may confidently declare he will be richly repaid for his perseverance, if he is anxious to become intimately acquainted with the various and afflicting maladies of which the author treats. To the work there are appended eight beautifully executed plates, illustrative of various kinds of deformity and spinal disease.

---

ART. IV.—*Manuel d'Anatomie Générale, Descriptive et Pathologique;* par J. F. MECKEL, Professeur d'Anatomie à l'Université de Halle. *Traduit de l'Allemand, et augmenté des Faits nouveaux dont la Science s'est enrichie jusqu'à ce jour;* par A. J. L. JOURDAN, Membre des Académies Royales de Médecine de Paris, &c. et G. BRESCHET, Professeur agrégè en Exercice, Chef des travaux Anatomiques de la Faculté de Médecine de Paris, &c. Tome troisième.—Paris: Baillière, 1825.

[Continued from page 254.]

IN pursuance of the plan which we announced in our last Number, we now proceed to give our readers some account of M. MECKEL's pathological views of the Nervous System; but we find this portion of his work much more imperfect than we had anticipated: in fact, with the exception of the anomalies of this system, and some considerations relative to the mode of union and regeneration of the nerves, their diseased conditions are wholly passed over. Thus we shall be enabled, in the course of the present paper, to give our author's opinions upon two of the subjects of which he treats,—namely, the Nerves, and the Diseases of the Bones.

Our author begins by describing the accidental lesions to which the nerves are subject, as naturally leading to an examination of their asserted power of regeneration. The extremities of a nerve which has been divided swell into a tubercle of a greater or less size: this tubercle is of a grey colour, and is often so solid and hard, that the scalpel, in cutting it, produces a sound similar to that caused by dividing cartilage. The size of this tubercle bears a relation to the quantity of surrounding cellular substance, and to the time that has elapsed since the wound; for it not only becomes larger by the lapse of time, but increases in hardness also. The portion of nerve situated below the wound is shrunk, and has lost its distinguishing colour. After amputations, this tubercle does not appear to be formed exactly at the extremity of the cut nerve; at least, VAN HORN found, an inch above the wound, that the nerves were confounded with the fleshy granulations arising from the muscles, without being able to distinguish them from the mass. A month, however, after the operation, they were of a reddish colour, both within as well as outwardly; and the tubercle, which was distinguishable from the extremity of the nerve by its white colour, was found situated still higher. The lower extremities of nerves are therefore destroyed, more or less, as those of other organs. It is further to be observed, that these tubercles are to be found upon the small, as well as upon the larger nerves, and they appear to last through the remainder of the life of the animal. If the lower portion of a nerve has not been removed, it will reunite with the upper portion; but physiologists are not agreed upon the nature of the substance by which they are united: some look upon it as a continuation of the nervous substance; others believe it to be formed of cellular tissue, or coagulated lymph, which never acquires the peculiar structure of nerve; from whence has arisen the contest relative to the possibility of regeneration in those organs.

There are two modes of becoming convinced of the regeneration of an organ: that is, by studying, in the first place, its original functions; and then by examining the nature of the substance which has been formed in its stead. The first method is very uncertain when applied to the nerves; since it is possible that the divided nerve may be replaced by anastomosing branches, and that a substance, similar in appearance to that which previously existed, may be sufficient to unite the two extremities, in such a manner that the functions of the part may be executed with its wonted regularity. The other method is more certain, though by no means free from fallacy.

Cruickshank, Haighton, Fontana, Michaelis, Munro, and Mayer, reasoning from very different grounds, have all come to the conclusion, that nerves cannot be regenerated perfectly. Arnemann, on the contrary, on the faith of numerous experiments, believes that they can. According to him, the cellular tissue, condensed by inflammation, is uniformly the means of union between the divided extremities of a nerve: it acquires sometimes the hardness of cartilage, but becomes united to those extremities in a very slow and gradual manner. Fontana believed that a true nervous substance was produced in some instances, where he had cut out a portion of the intercostal nerve, six lines in length, because nervous filaments passed through this intermediate substance. Michaelis also removed some portions of nerves, nine or ten lines in length, and found, at the termination of from two to eight weeks, that the divided extremities were united by a substance having a resemblance, nearly perfect, to the substance of the nerve. By the assistance of the microscope, he observed that the transition from the old nerve to the new was almost imperceptible. Mayer, having cut out portions of a nerve, to the extent of from one to two lines, found the two extremities reunited by filaments of a greater or less degree of fineness, which, like the real nervous substance, were not dissolved by nitric acid, but became harder, and consequently possessed one of the most essential qualities of this substance.

Haighton divided the eighth pair of nerves on one side of the neck of a dog, and six weeks afterwards he cut the nerve on the opposite side: at the expiration of six months, the animal was perfectly restored. But when the nerves on both sides were cut at the same time, or nearly so, all the animals perished. From these experiments he concluded, that death did not take place in the first instance, because the interval of six weeks was sufficient to permit the wound of the first nerve to be completely cicatrised. But, as it might be objected that the functions of the cut nerve might have been performed by others, which had increased in size, Haighton repeated the operation, by dividing at the same time the nerve on both sides; but the animal died, proving that the re-establishment of the functions had depended really on the reproduction of the nervous substance.

Arnemann has, however, rejected all the experiments favourable to the doctrine of the regeneration of nerve, in which they have been simply divided, without taking away any portion of them; in which case, he affirms there is no reproduction. But there is only a difference in degree in the mode of cicatrisation of wounds, either with or without loss of substance; since a simple division of parts does not unite *immediately*, the lymph which transudes from the divided edges giving rise to a new substance, which is the medium of union.

The experiments above mentioned seem to warrant our belief that this new substance, at first homogeneous in the wounds of all the organs, may at length become converted by degrees into the true nervous substance. Besides, it is not possible to prove that the new substance may not really be the same as that of the original nerve, although its external characters are distinct; since bone of a new formation differs from the old bone, both in structure and figure. Wounds of the brain, with loss of substance, also heal by means of a new formation, which does not resemble the part lost: it is more of a yellow colour, its texture is looser, it is softer,—sometimes even almost mucilaginous. Another circumstance very favourable to the cicatrisation of wounds of the brain, is the enlargement of the ventricle on the side opposite to the disease, and which is not productive of any risk, either to the life or even to the health of the patient. In the midst of the cerebral substance which is reproduced, a viscous matter is sometimes found, which Arnemann considers as principally formed of coagulable lymph, produced by the wound of the temporal muscle. It differs from the newlyformed cerebral substance, by its greater solidity, as well as being of a redder colour, and by being filled with newly-formed vessels.

The principal anomalies which the nervous system presents are—1st, its total or partial absence. The total absence of the nervous system is extremely rare, and can only be coincident with an imperfect development of the whole organisation, of which it is no doubt the result. A partial absence is more frequent. Most commonly a portion of the cerebral mass is found wanting; and it also not unfrequently happens that the spinal marrow has no existence, or, at least, that it only extends a very short distance. It sometimes happens the whole brain is wanting, though the spinal marrow is completely formed. It may be remarked that, whenever the fœtal brain has been found fully developed, the spinal marrow has never been wanting, and that the total absence of the brain has been more frequently met with in the female than the male. Particular nerves are seldom wanting, nor has the nervous system ever been found double; at least, unless the body is so also.

The second most remarkable anomaly of the nervous system, is its greater or less volume. The increase in the size of the nerves is extremely rare,—at least, whilst the nervous system is otherwise in a state of health. With regard to the opposite condition, that of their diminished size, it is not more common than other original vices of conformation. Atrophy of the nervous system is more frequently met with, and it may be either primitive or consecutive. That connected with the tabes dorsalis is an example of the former kind; atrophy of the optic nerve arising from the loss of sight, of the second; but then the nerve not only becomes smaller and thinner,—its texture also changes; it becomes harder, of a darker colour, and is less opaque.

In this place dropsy of the nervous system may be classed; and this condition is more frequently congenital than developed after birth. In the first instance, it occupies the whole system; in the second instance, it is confined to one part, most commonly to the brain.

3dly. Congenital anomalies of situation or form are extremely rare. Among those which are consecutive, may be distinguished rents *(dechirures)*, to which the brain is exposed, in consequence of effusion of blood in its substance.

Among the alterations of texture in this system may be remarked varieties in colour: this seldom occurs without being accompanied with other alterations of texture; nevertheless, the nervous system is more or less tinctured with a yellow colour in the jaundice.

A morbid hardness, or softness, of the nervous system may exist alone or together; that is, one portion may be harder, and another may be much softer, than usual. WEINHOLD assures us that the nerves are extremely soft, nay almost fluid, in typhous fever. The brain is sometimes softer, sometimes harder, in maniacal subjects. In epileptic patients, some points are occasionally found softer, and others harder, than in health. In hydrocephalus, the parietes of the brain are thinner and softer than natural.

New formations are by no means uncommon in the nervous system. Bone and fat are the only parts that are occasionally developed in the substance of the brain, and more rarely round the nerves. It is very common to meet with an ossific formation in the dura mater; but, on the contrary, it is not unusual to find new formations of different kinds develop themselves, either in the substance or on the surface of this system, more especially in the brain. Among these new formations may be enumerated encysted tumors, fibro-cartilaginous tumors of a yellowish-white colour, others having a resemblance to scrofulous tumors, and more rarely those of a fungous nature, abundantly supplied with blood, and firmly adhering to the brain; and, lastly, hydatids are not uncommon, and are to be found either in the ventricles or in the substance of the brain itself.

*Of the Morbid Conditions of the Bones.*

Primitive errors of conformation are not equally common to all bones; those of the cranium, and especially the occipital bone, offer the most frequent examples, and those of the extremities the fewest. These errors consist almost always in a suspension of the development of the bone, and it is probable that this bears some relation to the perfection of the brain, since these anomalies of the cranium coincide almost always with the incomplete development of that organ. Those affections of bone which are liable to occur at all periods of life, are *solutions of continuity*. This separation may be effected either by a cutting instrument or by a heavy blow: in the first case there is a wound, in the second a fracture. This may be either total or partial, transverse, oblique, or longitudinal: oblique fractures are the most common of all. After the growth of the body is completed, fractures may take place equally at all parts of the bone; but, when the epiphyses are not yet firmly connected to the body of the bone, it is very usual to see them detached, either by the effect of a mechanical injury, or in consequence of diseases which destroy the texture of the bone. In every case a cure may be effected, even when the bone is broken into fragments, or when a considerable portion of its substance is lost. Detached portions also will sometimes reunite, when placed in contact with the sound bone. The progress of the cure is absolutely the same as in the natural process of ossification: a gelatinous substance, which hardens little by little, is effused round the broken fragments and among them, and is converted into cartilage; in the interior of which, several ossific points afterwards appear, which unite together and with the broken parts, surrounding also those which had been completely detached. The fragments and splinters become rounded in a little time, so as not to wound the surrounding parts by their edges. To produce this new ossific formation, it is not necessary that the layers of bone separated from each other should have their corresponding faces in contact; for the cure takes place equally well when they are merely placed by the side of each other, provided no foreign body be interposed between them, and that they are kept in contact. Neither does it signify whether broken portions belong to the same or to a different bone; union takes place equally well; there is only anchylosis produced.

It seldom happens, if the subject is healthy, and the broken bones are placed in a proper situation, that the new bone is formed in too large a quantity: nevertheless, all fractures or wounds of bone do not recover in so complete a manner; neither are they always regenerated to the extent that has been lost. The causes of this difference are either mechanical or constitutional: among these latter causes must be reckoned old age, general debility, the effects of diseases, such as scurvy or rickets; or, lastly, the concentration of the activity of the plastic power in any other part of the body. Thus, fractures will not unite occasionally during pregnancy, or whilst the parent is giving suck. Sometimes these causes also dispose the callus to become soft, especially when the fracture has not been long consolidated. To the first class of impediments must be referred every thing which prevents the perfect contact of the broken surfaces, whether it be the original defect of coaptation, or a continual derangement of the particles by motion of the parts: hence it is that fractures of the ribs and of the patella are so often imperfectly united. In these cases an unnatural joint may be formed, and the limb or the part may become useless, or nearly so. The state of the parts is not, however, always the same in false joints; sometimes the fragments adhere to each other by an intermediate ligament; sometimes they remain separated, but are joined by a kind of capsular tissue, as in the movable joints; sometimes even muscular or tendinous fibres are to be met with between them.

Reproduction of bone goes on with much greater energy when a new bone is to be formed in the place of one which has been deprived of its life by some cause or other. It is the mortification of bone that constitutes the disease called *necrosis*, for the regeneration is only an accidental circumstance. The cylindrical bones possess this faculty in the greatest degree. When a portion of bone is dead, which does not always imply any great change, either in its form, colour, or chemical composition, it is detached from the sound portion, because nutrition does not extend beyond the line which separates the dead from the living parts, and absorption acts upon it with greater energy. At the same time the formation of new bone begins, it is the result of the enlargement of the vessels of the periosteum and cellular tissue in the vicinity. In proportion as the bone dies, a gelatinous fluid is effused over its surface, between it and the periosteum; this fluid thickens little by little, and is converted into true bone. At first it is cartilaginous, but in about twenty-four days after the commencement of the disease, ossific points are to be perceived in it; at length the new bone becomes consolidated with the old. As these two actions of mortification and reproduction usually proceed together, *pari passu*, the use of the limb is not generally lost to the patient. Even when the periosteum itself is deprived of life, this membrane is replaced by a new periosteum, which is formed from the neighbouring cellular membrane. In some respects, the newly-formed bone resembles that of the original formation; there are some differences, however. In length, hardness, and its connexion with the neighbouring parts, it is the same; but it has not the same form nor thickness. Generally it is of greater bulk, having been formed round the old bone; it is not so well shaped, nor are its fibres so regular; its surface is irregular, and marked with many asperities, and its cavity is generally obliterated.

The bone which has undergone necrosis never remains within the new bone: sometimes it disappears very gradually; at others it finds its exit altogether, or in small pieces, if not taken away by the surgeon. It generally forms for itself two or three round openings, which perforate the new bone entirely, and communicate with the surface by means of one or more fistulous openings. It may be remarked that these openings in the new bone seem to be coeval with its first formation; for, in the gelatinous matter effused, they are indicated by a few dry and opaque spots.

The death and reproduction of a cylindrical bone rarely extends beyond its body, its extremities remaining entire; and this is equally the case at every period of life. The necrosis does not include the whole thickness of the bone, for a small portion of its internal surface may alone be affected: in this case, the portions thrown off will not only be smaller, but they will also be rough; whereas, a bone which has mortified throughout its whole extent has a smooth surface.

The flat bones are often affected with necrosis, but generally they are not reproduced, or, at least, only in a very imperfect manner. If, however, regeneration does take place, it does not differ essentially from the process in the cylindrical bones.

The morbid increase or decrease in the volume of bones, may equally take place at any period of life. Sometimes the whole circumference of a bone increases in size, constituting *hyperostosis*; at others, the malady attacks only one particular point: this is called *exostosis*. In either case there is, or is not, an alteration of structure; the former of these circumstances is the most common. This alteration consists either in the bone being softer and more spongy than natural, or it may become heavier and more solid. *Osteosteatoma* bears some relation to exostosis, but it is probably attended with some change in the chemical composition of the bone. It very seldom happens that the bones diminish in volume, unless the other organs undergo a similar change from defect of nutrition.

The principal changes in the texture of bone are the following:—1st. Inflammation and its consequences, differing from the same affection in other parts from the slow progress which it makes. Thickening and swelling of a bone, and even exostosis, accompanied with a diminution of density, are unquestionably the results of the termination of inflammation. Suppuration of bone is called caries. 2d. A diminution of solidity and density. This may exist in different degrees, the least of which is that met with in cases of rickets. The bones of such patients are soft, spongy, flexible, and curved, either in those places where the muscles act upon them, the effects of which they are not able to resist, or where there is a weight to sustain; at the same time they receive too much blood, and the periosteum suffers analogous changes. The chemical composition of these bones is not the same throughout: sometimes there is too much phosphoric acid, at others too little; and the proportions between the animal substance and the earthy matter varies also greatly. These differences bear some relation to the intensity, or perhaps to the stage of the disease; but they prove, at least, that the rickets do not consist in an original defect of earthy substance. Children are the usual subjects of this complaint. The bones of the ricketty are too thick and too short; the head is more voluminous than natural, and the points of ossification in the bones of the cranium are strongly marked.

The condition above mentioned is carried to the highest point in that softened state of the bones called *osteo-sarcoma*: it then becomes soft and fleshy, so as to be easily cut with a knife; its cellular structure disappears, and it is converted into a homogenous substance; at the same time it swells to a greater or less extent, and is curved in proportion to its softness. This disease is most frequently met with in the female: the results which it causes arise from the bones yielding to the action of the muscles and the weight of the body.

The fragility of bone is a condition resembling the above in some measure, though it may be sometimes the consequence of an excess of earthy matter. This fragility is sometimes so great, that the slightest effort is sufficient to fracture the bone. This condition sometimes accompanies the softening of bone, but is more usually met with alone. Bone suffering under this disease does not lose its cellular structure; it even becomes more strongly marked. The principal causes of this condition are diseases of a long continuance, which attack the whole system, such as scurvy, cancer, or syphilis.

### *Of the Articulations.*

The morbid changes occurring in the joints are principally the rupture, distention, or relaxation of the ligaments, giving rise to luxations. When the bone does not return of its own accord, or is not restored by art to its original situation, a new joint is formed, and the original one is effaced. The bone against which the luxated one has placed itself forms a small superficial cavity, which is at first covered with periosteum, but afterwards obtains a covering of cartilage, and which has a border more or less turned outwards; at the same time the head of the luxated bone becomes flatter and more unequal than before, and loses altogether, or in part, its cartilaginous covering, because the muscles press it more forcibly against the corresponding bone. Sometimes a cavity is formed in the dislocated bone, which corresponds with a projection growing from the surface of the neighbouring one. Immovable articulations are sometimes separated, or more loosely united, by the effect of a primitive error of conformation. Hydrocephalus, and the want of union in the symphysis pubis, afford examples of this latter affection. The former, with the exception of the separation of the symphysis pubis in the latter period of pregnancy, only takes places in consequence of mechanical violence, either violent and sudden, or slow and constantly increasing.

The excess of solidity in a joint is called *anchylosis*. This may be either false or true, and a loss of motion in the joint is the necessary consequence. In this case, either the ligamentous fibres become ossified, or a formation of ossific matter takes place beneath them, which unites the two bones in the manner of a bridge, or else they are united throughout the whole extent of their corresponding surfaces, so that the cartilage with which they were covered, and the compact substance of the bone, has disappeared, and nothing is found but a spongy substance, extending throughout the whole length of the bone. This last affection is the consequence of inflammation and suppuration of the extremities of bones. Sometimes, without any apparent cause, a tendency to ossification is found to exist in several of the joints, or even in them all; a general rigidity of the whole body is the consequence.

### *On accidental Ossification.*

The accidental formation of bone is a phenomenon that very frequently occurs, but which, generally speaking, takes place only in the latter period of life: it is common in certain parts of the arterial system,—that is, in the internal membrane of the aorta; but it is not much less rare in the serous membranes. Accidental formations of bone also occasionally take place in the female, either in the uterus, the ovaria, or the thyroid gland. These ossific formations present themselves under two different forms: sometimes it forms one continuous substance with the parts in the midst of which it grows; at others, it forms a separate part, having no connexion with the substance from which it arises but by means of its roots, and from which it finally becomes separated. The first species of ossification is found principally in the vascular system, and in several parts of the serous system. Those of the latter kind are found in the bursæ mucosæ, or in the synovial membranes; nevertheless, they are sometimes met with in some of the serous membranes, and especially in the tunica vaginalis testis.

The first-mentioned ossific formations present patches of a greater or less extent, which scarcely project from the surface of the parts to which they are affixed. The latter constitute rounded pediculated bodies, which are especially met with in those joints subjected to frequent shocks: they are sometimes single, at others very numerous, and always communicate, either at one time or other, with the synovial membrane.

[To be continued.]

